# Nomogram for predicting early olfactory dysfunction in obstructive sleep apnea-hypopnea syndrome: a multicenter-based study

**DOI:** 10.3389/fneur.2025.1635012

**Published:** 2025-09-17

**Authors:** Qingchun Pan, Qi Yang, Jing Huang, XueQin Mi, Bei Li

**Affiliations:** ^1^Department of Otolaryngology, Head and Neck Surgery, Affiliated Hospital of North Sichuan Medical College, Nanchong, China; ^2^Department of Otolaryngology, Head and Neck Surgery, Chengdu Sixth People's Hospital, Chengdu, China

**Keywords:** sleep apnea, obstructive, olfactory function, sleep structure, nomogram

## Abstract

**Objective:**

To develop and validate a clinical prediction model for olfactory dysfunction in patients with obstructive sleep apnea-hypopnea syndrome (OSAHS), evaluating the combined predictive value of polysomnography (PSG) parameters and clinical symptoms.

**Methods:**

We retrospectively analyzed 546 OSAHS patients, including 420 from the Affiliated Hospital of North Sichuan Medical College were [randomly divided into training (*n* = 294) and internal validation (*n* = 126) sets], and 126 from the Sixth People's Hospital of Chengdu (external validation set). All patients underwent overnight PSG for sleep parameter assessment and Sniffin' Sticks tests for olfactory evaluation. Predictors were selected using LASSO regression with subsequent logistic regression modeling, followed by nomogram construction. Model performance was assessed through ROC analysis, calibration curves and DCA curves.

**Results:**

Among 546 enrolled patients, with OSAHS were included in this study. The overall olfactory dysfunction incidence was 38.64% (211/546). Multivariable analysis identified seven independent predictors: gender, age, AHI, N3%, REM%, TS90%, and MoCA. The predictive efficacy AUC of the training set model was 0.832 (95% CI: 0.784–0.880); good calibration (slope = 0.89, Hosmer–Lemeshow *P* = 0.41); and clinical utility across threshold probabilities of 0.06–0.97.

**Conclusion:**

Our prediction model constructed based on gender, age, AHI, N3%, REM%, TS90%, and MoCA can effectively identify OSAHS patients at high risk for olfactory dysfunction. With robust discrimination and calibration, this tool provides a clinically useful, non-invasive method for early risk stratification and intervention planning.

## Introduction

Obstructive sleep apnea hypopnea syndrome (OSAHS) is a prevalent chronic sleep-related breathing disorder marked by recurrent upper airway collapse, leading to apnea or hypopnea during sleep (1). Global epidemiological data indicate that OSAHS affects 9%−38% of adults and is strongly associated with multisystem complications, including cardiovascular and metabolic diseases as well as neurocognitive impairment (2). In recent years, sensory dysfunction in OSAHS has garnered increasing attention. Notably, olfactory dysfunction has emerged as an interdisciplinary research focus due to its implications for nutritional imbalance and its potential role as an early marker for neurodegenerative diseases (3). Studies report that 38 to 65% of OSAHS patients exhibit olfactory impairment, which not only contributes to appetite loss and nutritional deficiencies, but also elevates the risk of accidents due to the diminished odor detection (4). Importantly, progressive olfactory decline may precede typical symptoms of OSAHS symptoms and has been been linked to the pathological progression of Alzheimer's disease and Parkinson's disease (5). Therefore, studies investigating the risk identification of olfactory dysfunction in patients with OSAHS are of great importance, though several limitations remain in the existing research. First, studies often examine isolated parameters, focused solely on Apnea Hypopnea Index (AHI), examined only hypoxia indices, and considered only sleep architecture parameters; second, most existing prediction models use unidimensional indicators, such as Dong et al.'s demographic-only approach or Shin et al.'s AHI-only analysis, failing to incorporate the interplay of multimodal PSG parameters and demographic factors; third, methodological constraints, including small single-center samples and traditional regression approaches vulnerable to multicollinearity (3–8).

Evidence suggests that age and gender are independent influencing factors for olfactory dysfunction in OSAHS (8). Additionally, studies propose that sleep disruption, nocturnal hypoxia and daytime sleepiness impair activity in brain regions involved in odor processing (such as thalamus, prefrontal cortex, posterior parietal cortex, and hippocampus), as well as the hippocampus in the brain that processes odors, contributing to olfactory deficits in OSAHS (9). PSG, the gold standard for evaluating sleep in OSAHS, provides objective measures of sleep architecture and hypoxia (10). Multiple studies confirmed that PSG-derived metrics—such as the AHI—correlate strongly with olfactory dysfunction, with oxygen saturation below 90% identified as an independent risk factor (8, 11). Intriguingly, recent findings highlight a significant association between the Montreal Cognitive Assessment (MoCA) score and olfactory function, suggesting that the central cognitive pathway may modulate OSAHS-related olfactory impairment. This supports the rationale for constructing a multi-dimensional prediction model Study Design and Innovation (7).

This multicenter retrospective study integrates multimodal PSG parameters, clinical symptoms and multi-dimensional indexes (such as psychological assessment). Variable selection employed LASSO regression with 10-fold cross-validation. We chose LASSO over ridge regression for its variable selection properties, particularly valuable given our exploratory aims with multiple correlated PSG parameters. The optimal λ was selected using the one standard error rule to balance model complexity and predictive accuracy. Sensitivity analysis across λ values (0.01–0.05) confirmed stable variable selection. By incorporating a nomogram visualization tool, we transform complex models into an intuitive scoring system, enabling real-time individual risk stratification to enhance clinical interpretability and decision-making. This approach offers a non-invasive tool for early identification of olfactory dysfunction in OSAHS patients.

## Materials and methods

### Participants and study design

This study retrospectively enrolled patients diagnosed with OSAHS by PSG who visited the Department of Otorhinolaryngology Head and Neck Surgery at the Affiliated Hospital of North Sichuan Medical College and the Sixth People's Hospital between January 2020 and March 2024.

Inclusion criteria: (1) met the diagnostic criteria of OSAHS according to the International Classification of Sleep Disorders Third Edition (ICSD-3) (12), defined as an AHI ≥ 5 times/h with predominantly obstructive events (apneas or hypopneas); (2) age ≥ 18 years old; (3) capable of cooperating to complete olfactory function testing.

Exclusion criteria: (1) history of nasal surgery, chronic sinusitis, deviated nasal septum, atrophic rhinitis, nasal polyps, or acute exacerbation of allergic rhinitis; (2) comorbid neurodegenerative disorders (e.g., Alzheimer's disease, Parkinson's disease) or history of head trauma; (3) pregnant or lactating women; (4) use of medications that may affect olfaction (e.g., glucocorticoids, antihistamines) within 1 month prior to enrollment.

**Figure 1:**
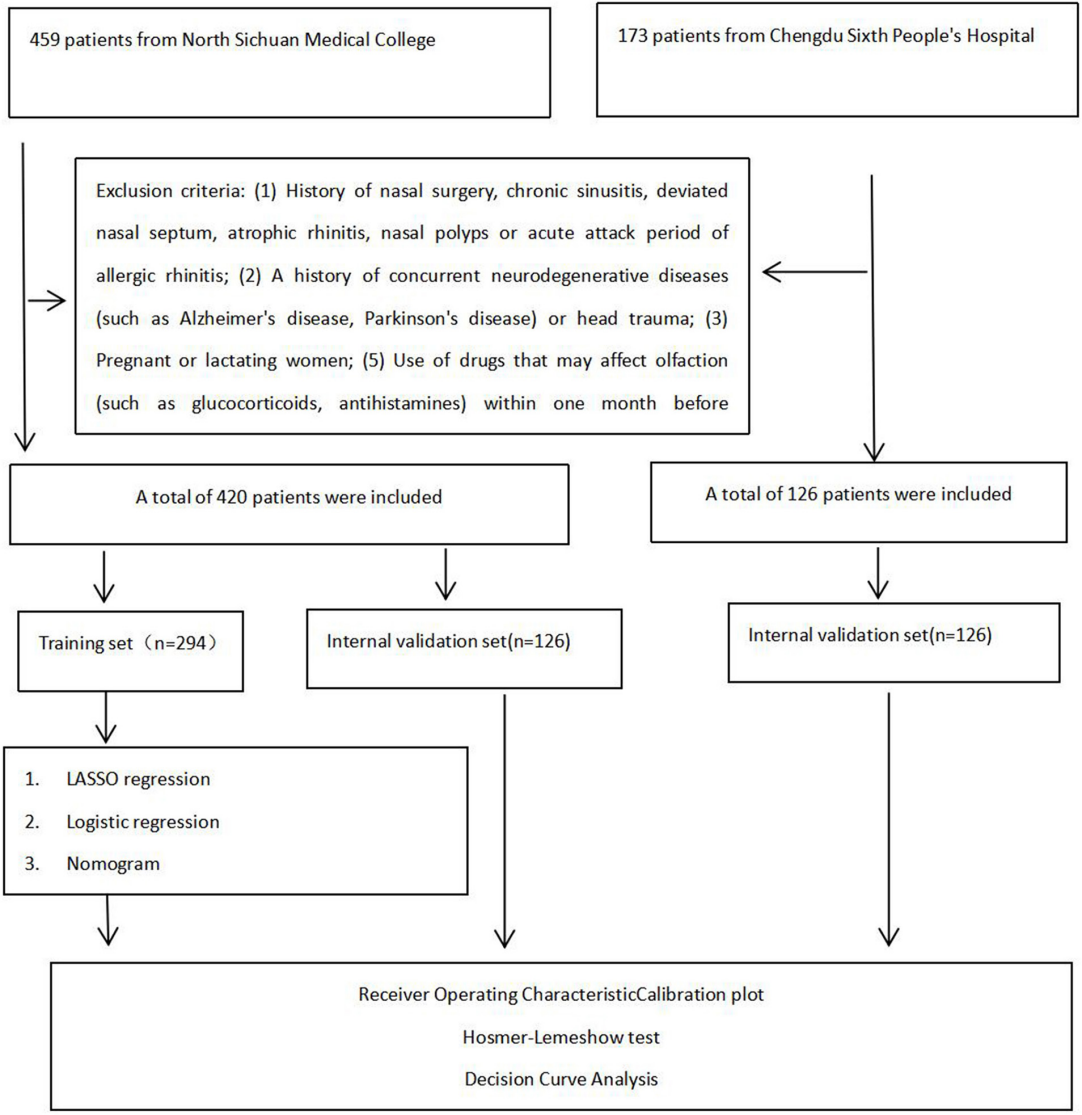
Flowchart diagram of research strategy in the Affiliated Hospital of North Sichuan Medical College and Chengdu Sixth People's Hospital.

This study protocol was approved by the Ethics Committee of both participating hospitals (Approval No.: 2020ER035-1), and written informed consent was obtained from all participants. The flowchart diagram of the research strategy is presented in Figure 1.

This study employed the Logistic regression sample size calculation based on the principle that the number of outcome events at least 10 times the number of independent variables. Following predictive modeling guidelines and bias assessment tools, a minimum of 10 events per independent variable was required (13). With olfactory dysfunction as the dependent variable and a maximum of 13 potential predictors identified through literature review, and given an estimated 40% prevalence of olfactory disorders in OSAHS patients, the minimum required sample size was calculated as 13 × 10÷40% = 325. Accounting for potential invalid samples with a 20% buffer, we ultimately enrolled 546 participants, we ultimately enrolled 546 participants. These included 420 patients from the Affiliated Hospital of North Sichuan Medical College [randomly allocated to training (*n* = 294) and internal validation (*n* = 126) sets] and 126 patients from Chengdu Sixth People's Hospital (external validation set).

Collect the patients' gender, age, educational level, BMI, smoking status, alcohol consumption (heavy drinking defined as >5 years with daily ethanol intake ≥40 g for men/≥20 g for women), and family history of OSAHS. Comorbidiasis such as hypertension, hyperlipidemia, hyperglycemia, insomnia was evaluated by the Insomnia Severity Index (ISI), daytime sleepiness was evaluated by the Epworth Sleepiness Scale (ESS), and the MoCA assessment of patients' cognitive level, olfactory function assessment, and PSG detection. Hypertension (diagnosed as systolic BP ≥ 140 mmHg or diastolic BP ≥ 90 mmHg) (14). Hyperlipidemia (fasting total cholesterol ≥ 5.72 mmol/L or triglycerides ≥ 1.7 mmol/L) (15). Hyperglycemia (fasting glucose ≥ 7.0 mmol/L or postprandial ≥ 11.1 mmol/L) (16). ISI evaluated the insomnia of patients using 7 items, with a total score ranging from 0 to 28 points. The higher the score, the more severe the insomnia (17). ESS evaluated the daytime sleepiness of patients using eight questions, with a total score ranging from 0 to 24. The higher the score, the more severe the daytime sleepiness (18). The assessment of cognitive memory level and the group use of MoCA to evaluate cognitive function have a total score of 30 points. An additional one point is added if the years of education are ≤ 12 years to correct the bias (19).

### Polysomnography (PSG)

Polysomnography monitoring was performed using the Somte E system (Compumedics, Australia) with continuous recording of electroencephalogram, submental electromyogram, oronasal airflow, thoracoabdominal respiratory effort, oxygen saturation, and body positioning according to the American Academy of Sleep Medicine (AASM) standards. Polysomnographic parameters included:AHI, total sleep time (TST), sleep efficiency (SE), oxygen desaturation index (ODI), wake after sleep onset (WASO), and sleep stage proportions (N1, N2, N3, REM%) calculated as percentages of TST, with hypoxemia defined as SpO_2_ < 90%.

### Olfactory function assessment

Olfactory testing was performed using the Sniffin' Sticks kit (Burghart GmbH, Germany) in a controlled environment (22 ± 1 °C; 50 ± 5% humidity) by certified technicians. Threshold test threshold (T): the odor perception threshold was detected using the concentration gradient of phenylethyl alcohol (1:2 dilution, 16 steps). Detection thresholds were determined via ascending forced-choice paradigm, defined as the lowest concentration achieving three consecutive correct odor identifications, scored on a 0–16 scale. Discrimination test discrimination (D): odor discrimination testing employed 16 triplet sets (one target; two distractors per trial). Patients were required to identify the target odor, with the number of correct identifications recorded as the discrimination score (0–16 points). Identification test identification (I): participants were required to identify 16 familiar odors (e.g., coffee, mint) by selecting the correct name from four candidate options. The number of correct responses determined the recognition score (0–16). The total score TDI = T + D + I. Normal reference values: TDI score >30 (normal olfactory function), 28–30 points (mild impairment), 16–27 (moderate impairment), ≤ 15 points (severe impairment) (20). In this study, the group with a TDI score ≤ 30 was categorized as the olfactory disorder (OD) group, while the group with a TDI score >30 was classified as the Non-olfactory disorder (Non-OD) group.

### Statistical analysis

Data analysis was conducted in this study using SPSS 26.0 and R 4.3.1 software. The distribution of continuous variables was assessed using the Shapiro–Wilk test. Normal distributed data were presented as mean ± standard deviation (±s), and ANOVA was applied to compare groups. Non-normal data were described as median quartiles [M (P25, P75)], and the differences between groups were tested by the Kruskal–Wallis *H* test; Categorical variables were presented as frequency (percentage), and the χ^2^ test or Fisher's exact test was used for comparison between groups. The prediction model was constructed through three stages: our predictive modeling approach employed a direct LASSO (Least Absolute Shrinkage and Selection Operator) regression framework with 10-fold cross-validation to simultaneously perform variable selection and regularization. This advanced technique automatically identifies the most predictive variables while handling multicollinearity through coefficient shrinkage, with the optimal λ value determined by minimizing cross-validation error. The LASSO-selected variables were then incorporated into a multivariate logistic regression model to estimate odds ratios (ORs) with 95% confidence intervals (CIs). Model validation was assessed through three aspects: discrimination (ROC curve AUC), calibration (Bootstrap 1000 resampling calibration curve and Hosmer–Lemeshow test), and clinical efficacy [decision curve analysis (DCA)]. Meanwhile, the linear assumption of continuous variables was evaluated using the Box–Tidwell test. The Benjamini–Hochberg method was employed to control the false discovery rate (FDR) in multiple comparisons (FDR < 0.05). The nomogram was constructed using the “rms” package in R language. All analyses were performed on the complete dataset without any imputation.

## Results

### Baseline characteristics

A total of 546 patients were enrolled in this study, of whom 38.64% (211/546) had OD. Of the 420 patients at the Affiliated Hospital of North Sichuan Medical College, 294 were randomly allocated to the training set, with 38.10% (112/294) exhibiting olfactory disorders. Of the 126 patients in the internal validation set, 38.10% (48/126) exhibited OD. A total of 126 patients with OSAHS from the Sixth People's Hospital of Chengdu served as the external validation set, with 40.48% (51/126) exhibiting olfactory disorders. No significant differences were observed in comparisons of data across the training, internal validation, and external validation sets (*P* > 0.05), as shown in Table 1.

**Table 1:** Baseline characteristics of patients in the training set and the internal validation set [$\bar{x}\pm$*s* or M (P_25_, P_75_)].

**Variable**	**Study population (*****n*** = **546)**		**Training set (*****n*** = **294)**		**Internal validation set (*****n*** = **126)**		**External validation set (*****N*** = **126)**		***X^2^/F/H***	***P*-value**
	**Non-OD (*****n*** = **335)**	**OD (*****n*** = **211)**	**Non-OD (*****n*** = **182)**	**OD (*****n*** = **112)**	**Non-OD (*****n*** = **78)**	**OD (*****n*** = **48)**	**Non-OD (*****n*** = **75)**	**OD (*****n*** = **51)**		
**Gender (** ***n*** **, %)**										
Male	78 (23.28)	93 (44.08)	43 (23.63)	47 (41.96)	17 (21.79)	22 (45.83)	18 (24.00)	24 (47.06)	0.314	0.855
Female	257 (76.72)	118 (55.92)	139 (76.37)	65 (58.04)	61 (78.21)	26 (54.17)	57 (76.00)	27 (52.94)		
**Age (** ***n*** **, %)**										
<60	254 (75.82)	118 (55.92)	132 (72.53)	62 (55.36)	64 (82.05)	28 (58.33)	58 (77.33)	28 (54.90)	2.008	0.366
≥60	81 (24.18)	93 (44.08)	50 (27.47)	50 (44.64)	14 (17.95)	20 (41.67)	17 (22.67)	23 (45.10)		
Years of education [M (P25, P75), years]	7 (5, 9)	7 (5, 9)	7 (5, 9)	7.5 (5, 9)	7 (5, 9)	7 (5, 9)	7 (5, 9)	8 (5, 10)	1.419	0.492
BMI (x ± s, kg/m^2^)	30.94 ± 2.93	31.06 ± 3.07	31.06 ± 2.78	30.55 ± 2.92	30.95 ± 3.25	31.84 ± 3.14	30.63 ± 2.96	31.45 ± 3.16	0.89	0.411
**History of smoking (** ***n*** **, %)**										
No	265 (79.10)	160 (75.83)	144 (79.12)	88 (78.57)	60 (76.92)	33 (68.75)	61 (81.33)	39 (76.47)	1.552	0.46
Yes	70 (20.90)	51 (24.17)	38 (20.88)	24 (21.43)	18 (23.08)	15 (31.25)	14 (18.67)	12 (23.53)		
**Alcohol abuse (** ***n*** **, %)**										
No	295 (88.06)	178 (84.36)	158 (86.81)	96 (85.71)	69 (88.46)	39 (81.25)	68 (90.67)	43 (84.31)	0.339	0.844
Yes	40 (11.94)	33 (15.64)	24 (13.19)	16 (14.29)	9 (11.54)	9 (18.75)	7 (9.33)	8 (15.69)		
**Hypertension (** ***n*** **, %)**										
No	253 (75.52)	154 (72.99)	137 (75.27)	83 (74.11)	58 (74.36)	34 (70.83)	58 (77.33)	37 (72.55)	0.216	0.898
Yes	82 (24.48)	57 (27.01)	45 (24.73)	29 (25.89)	20 (25.64)	14 (29.17)	17 (22.67)	14 (27.45)		
**Diabetes (** ***n*** **, %)**										
No	274 (81.79)	165 (78.20)	152 (83.52)	92 (82.14)	61 (78.21)	35 (72.92)	61 (81.33)	38 (74.51)	2.939	0.230
Yes	61 (18.21)	46 (21.80)	30 (16.48)	20 (17.86)	17 (21.79)	13 (27.08)	14 (18.67)	13 (25.49)		
**High blood lipid (** ***n*** **, %)**										
No	262 (78.21)	159 (75.36)	141 (77.47)	82 (73.21)	64 (82.05)	40 (83.33)	57 (76.00)	37 (72.55)	2.817	0.244
Yes	73 (21.79)	52 (24.64)	41 (22.53)	30 (26.79)	14 (17.95)	8 (16.67)	18 (24.00)	14 (27.45)		
**Family history of OSAHS (** ***n*** **, %)**										
No	287 (85.67)	173 (81.99)	155 (85.16)	91 (81.25)	69 (88.46)	40 (83.33)	63 (84.00)	42 (82.35)	0.638	0.727
Yes	48 (14.33)	38 (18.01)	27 (14.84)	21 (18.75)	9 (11.54)	8 (16.67)	12 (16.00)	9 (17.65)		
AHI (x ± s, time/h)	34.55 ± 5.09	37.41 ± 5.03	34.80 ± 5.13	37.96 ± 5.26	34.37 ± 5.15	37.00 ± 5.03	34.12 ± 4.95	36.61 ± 4.42	1.456	0.234
TST (x ± s, min)	409.72 ± 21.06	409.17 ± 19.11	409.73 ± 20.49	408.20 ± 19.07	411.10 ± 23.68	412.02 ± 19.72	408.27 ± 19.69	408.61 ± 18.74	0.810	0.446
SE [M (P25, P75), %]	86 (79, 92)	85 (79, 91)	86 (79, 92)	84.5 (78, 91.5)	88 (80, 92)	86 (79, 89.5)	85 (77, 91)	85 (79, 95)	0.881	0.644
TS90%[M (P25, P75), %]	3.42 (2.39, 4.46)	4.15 (3.19, 5.42)	3.36 (2.36, 4.48)	4.22 (2.79, 5.42)	3.32 (2.21, 3.97)	3.78 (3.37, 5.47)	3.87 (2.88, 4.63)	4.4 (3.37, 5.36)	5.330	0.070
LSaO_2_ (x ± s, %)	82.54 ± 6.20	80.62 ± 5.80	82.27 ± 6.45	80.69 ± 5.22	82.94 ± 6.16	80.38 ± 7.22	82.79 ± 5.66	80.68 ± 5.63	0.143	0.867
WASO (x ± s, min)	44.94 ± 4.09	45.12 ± 4.01	44.77 ± 4.00	45.27 ± 4.01	45.37 ± 4.32	44.44 ± 3.77	44.89 ± 4.09	45.45 ± 4.22	0.066	0.937
N1% (x ± s, %)	33.15 ± 7.32	36.21 ± 7.33	33.39 ± 7.92	36.19 ± 7.55	33.05 ± 6.57	35.77 ± 7.48	32.69 ± 6.57	36.65 ± 6.8	0.113	0.894
N2% (x ± s, %)	43.39 ± 7.99	45.09 ± 7.42	43.14 ± 8.01	45.38 ± 7.65	43.11 ± 7.58	44.39 ± 7.85	44.29 ± 8.39	45.09 ± 6.57	0.546	0.580
N3% (x±s,% )	7.40 ± 3.48	5.96 ± 3.09	7.36 ± 3.56	5.93 ± 2.98	7.60 ± 3.37	6.09 ± 3.29	7.29 ± 3.43	5.92 ± 3.21	0.254	0.775
REM% (x ± s, min)	13.87 ± 5.72	11.50 ± 5.22	14.04 ± 5.97	11.40 ± 5.73	14.05 ± 5.2	11.52 ± 4.65	13.25 ± 5.62	11.71 ± 4.6	0.276	0.759
ESS [M (P25, P75), min]	7 (6, 8)	7 (6, 8)	7 (6, 8)	7 (6, 8)	7 (6, 8)	7 (6, 8)	7 (6, 8)	7 (6, 8)	3.290	0.193
ISI [M (P25, P75), min]	10 (9, 11)	10 (9, 11)	10 (9, 12)	10 (9, 11)	10 (9, 11)	10 (8.5, 11)	10 (9, 11)	10 (9, 12)	1.258	0.533
MOCA [M (P25, P75), min]	26 (24, 27)	23 (21, 26)	26 (23, 28)	24 (22.5, 25.5)	26 (25, 28)	24 (22, 26)	26 (24, 27)	23 (21, 26)	0.504	0.777

OSAHS, obstructive sleep apnea-hypopnea syndrome; BMI, body mass index; AHI, apnea-hypopnea index; TST, total sleep time; SE, sleep efficiency; NREM, non-rapid eye movement sleep; REM, rapid eye movement sleep; WASO, waking time after falling asleep; N1/TST%, the percentage of N1 stage sleep in TST; N2/TST%, the percentage of N2 stage sleep in TST; N3/TST%, the percentage of N3 stage sleep in TST; REM/TST%, the percentage of REM stage sleep in TST; ESS, Epworth sleepiness scale; ISI, insomnia severity index scale; MMSE, mini mental state examination.

### Univariate analysis of olfactory disorders in patients with OSAHS in the training set

**Figure 2:**
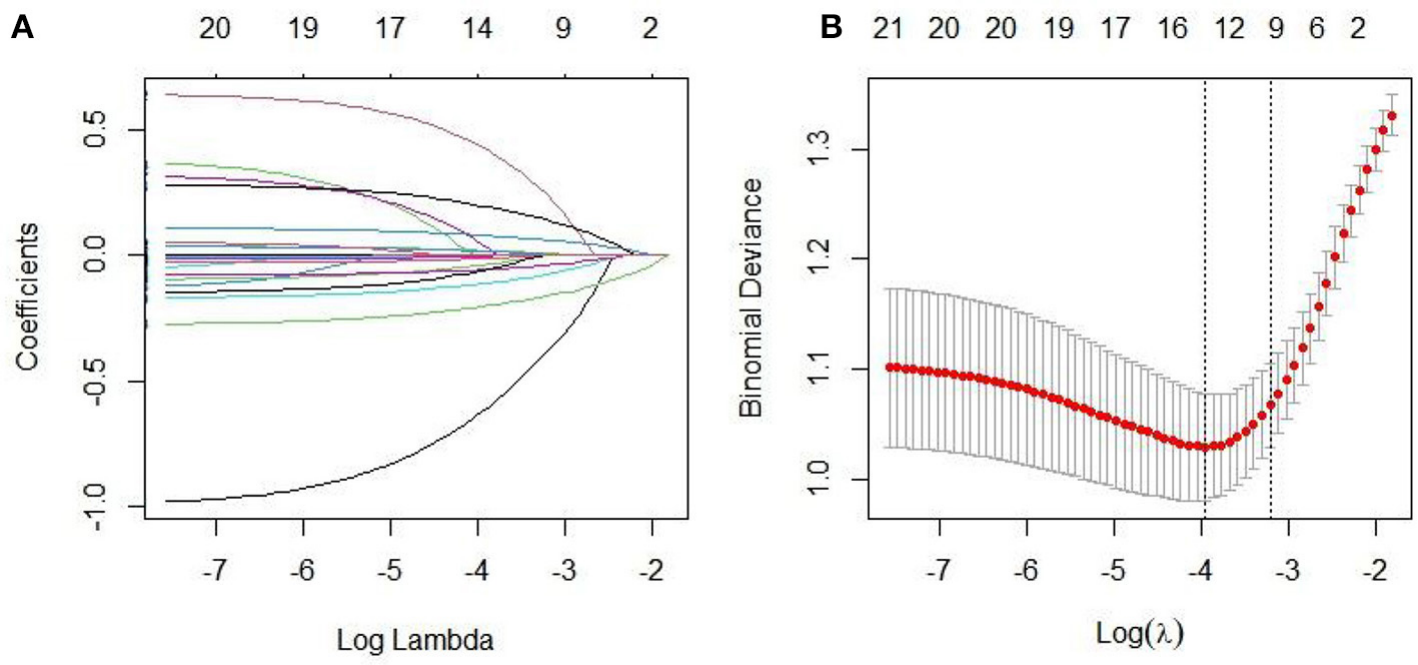
**(A, B)** screens the predictor variables based on LASSO regression.

A total of 294 patients with OSAHS were enrolled in the training set, of whom 112 experienced recurrence, resulting in a recurrence rate of 38.10%. LASSO regression was used to screen for non-zero coefficient predictors in 23 variables, including gender, age, education level, BMI, smoking, excessive drinking, familial OSAHS, and comorbidities such as hypertension, hyperlipidemia, hyperglycemia, ESS, MoCA, AHI, LSaO_2_, N1/N2/N3/REM, TS90%, etc. The 10-fold cross-validation method was employed to identify the optimal λ parameter, with the aim of reducing the number of variables while ensuring model performance. Following screening, 10 key predictors with non-zero coefficients were identified, including male, age, AHI, LSaO_2_, N1, N2, N3, REM, TS90%, and MoCA. The LASSO regression path plot and visualization of variable selection for the best model are depicted in Figures 2A, B, respectively.

### Logistic regression model for OD in patients with OSAHS training set

With olfactory disorders occurring in patients with OSAHS as the dependent variable and the LASSO regression method employed to identify 10 key factors as independent variables, a logistic regression model was constructed. The results demonstrated that gender, age, AHI, N3, REM, TS90, and MoCA were independent associated factors of olfactory disorders in patients with OSAHS (*P* < 0.05), as shown in Tables 2 and 3.

**Table 2:** Assignment methods of independent variables.

**Variable**	**Value**
Gender	Male = 1; female = 2
Age	<60 = 1; ≥60 = 2
AHI	Continuous variable
LSaO_2_	Continuous variable
N1%	Continuous variable
N2%	Continuous variable
N3%	Continuous variable
REM%	Continuous variable
TS90%	Continuous variable
MoCA	Continuous variable

**Table 3:** Multivariate logistic regression analysis of OD in patients with OSAHS in the training set.

**Variable**	***B***	**S.E**.	**Wald**	***P*-value**	**OR**	**95% CI**
Gender	−0.844	0.327	6.644	0.010	0.430	0.226–0.817
Age	0.719	0.314	5.251	0.022	2.052	1.110–3.796
AHI	0.107	0.030	12.95	0.000	1.113	1.050–1.180
LSaO_2_	−0.029	0.026	1.315	0.251	0.971	0.923–1.021
N1%	0.034	0.021	2.533	0.112	1.035	0.992–1.079
N2%	0.032	0.019	2.741	0.098	1.033	0.994–1.072
N3%	−0.170	0.049	12.137	0.000	0.843	0.766–0.928
REM%	−0.078	0.028	8.063	0.005	0.925	0.876–0.976
TS90%	0.267	0.102	6.818	0.009	1.306	1.069–1.596
MoCA	−0.255	0.055	21.919	0.000	0.775	0.696–0.862
Constant	3.250	3.058	1.130	0.288	25.785	–

Among 10 variables initially selected by LASSO regression, only seven retained statistical significance (*p* < 0.05).

**Figure 3:**
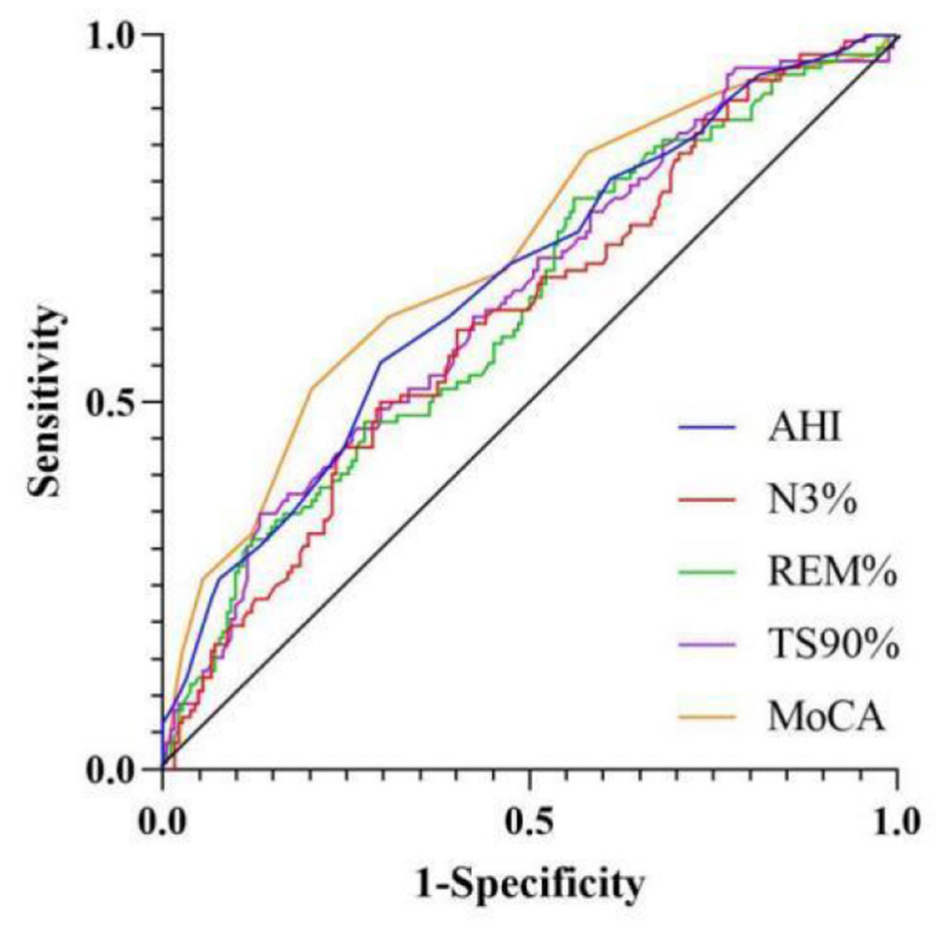
ROC curve analysis of related factors.

ROC curve analysis of related variables was conducted for AHI, N3, REM, TS90%, and MoCA. The results showed that the area under the curve (AUC) was 0.660 (95% CI 0.596–0.724), 0.613 (95% CI 0.548–0.679), 0.626 (95% CI 0.560–0.691), 0.638 (95% CI 0.573–0.703), and 0.698 (95% CI 0.636–0.760), respectively, as shown in Tables 4 and Figure 3.

**Table 4:** ROC curve analysis of related factors.

**Variable**	**AUC**	**95% CI**	***P*-value**	**Sensitivity**	**Specificity**	**Threshold**
AHI	0.660	0.596–0.724	< 0.001	55.4	70.3	37.5
N3%	0.613	0.548–0.679	0.001	50.0	70.3	5.315
REM	0.626	0.560–0.691	< 0.001	77.7	44.0	14.615
TS90	0.638	0.573–0.703	< 0.001	34.8	86.8	5.02
MoCA	0.698	0.636–0.760	< 0.001	51.8	79.7	23.5

**Figure 4:**
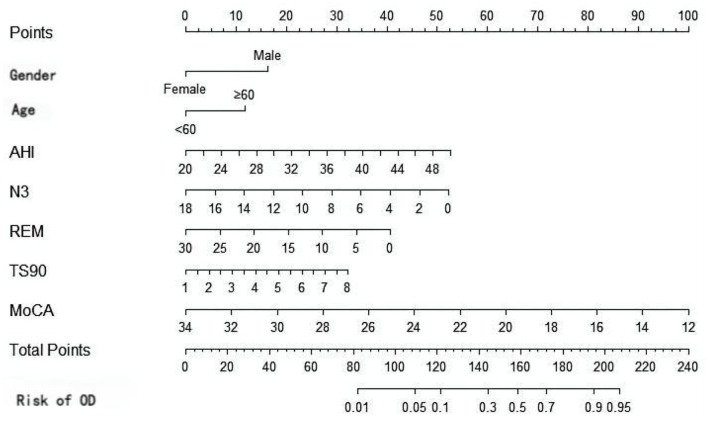
Nomogram prediction model.

Based on the results of multivariate Logistic regression analysis, gender, age, AHI, N3, REM, TS90%, and MoCA were included to construct a nomogram model for risk prediction of olfactory disorders in OSAHS patients in the training set. The nomogram (Figure 4) provides a clinically applicable visual tool for individualized risk estimation of olfactory dysfunction in OSAHS patients. Key predictors are assigned weighted points along the top axis based on their contribution: age≥60 years (25 points), male gender (30 points), AHI >40 events/h (40 points), N3% < 6% (20 points), REM% < 10% (15 points), TS90% >5% (25 points), and MoCA score ≤ 24 (30 points). To implement this tool: (1) locate each patient's specific values on the corresponding variable axes, (2) sum all assigned points on the “Total Points” axis, and (3) project this sum to the bottom “Risk” axis to obtain the predicted probability.

### Verification of the model

**Figure 5:**
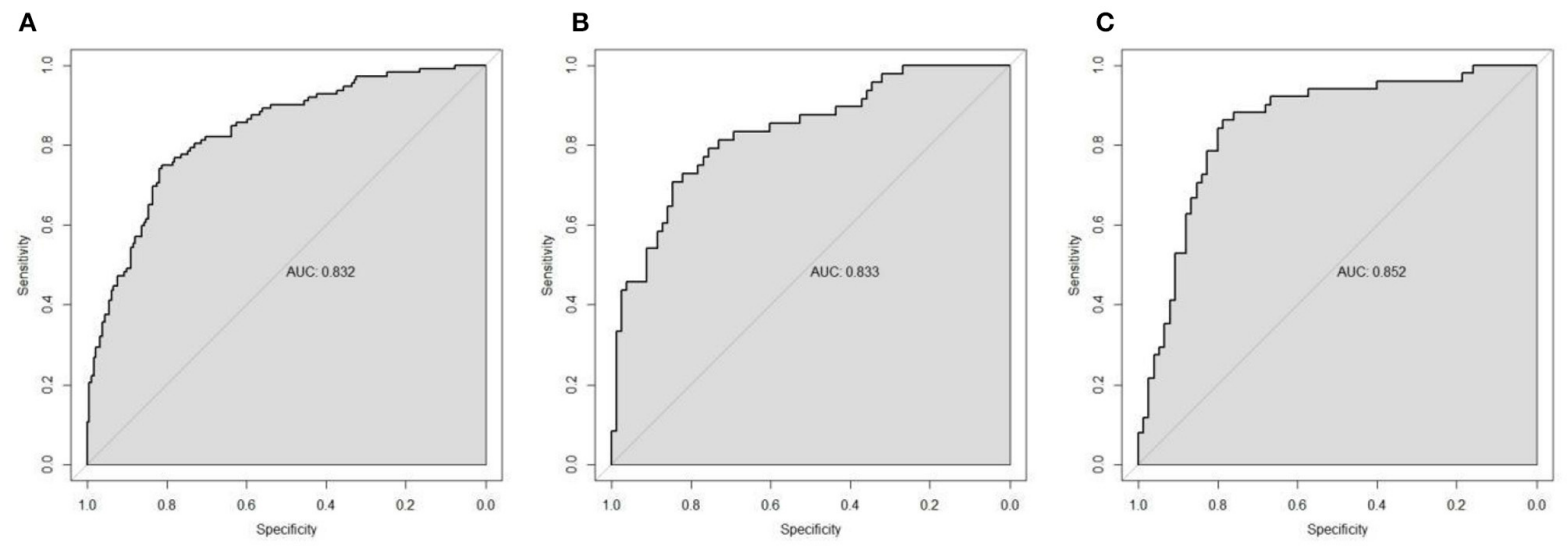
The receiver operating characteristic (ROC) curves of the model in the training set **(A)**, internal validation set **(B)**, and external validation set **(C)**.

The discrimination of the nomogram model was evaluated through the ROC curve. The results demonstrated good discriminatory performance in predicting the presence of olfactory disorders in patients with OSAHS. The AUC of the training set was 0.832 (95% CI, 0.784–0.880; Figure 5A), the AUC of the internal validation set was 0.833 (95% CI, 0.760–0.906; Figure 5B), the AUC of the external test set was 0.852 (95% CI, 0.782–0.921; Figure 5C).

**Figure 6:**
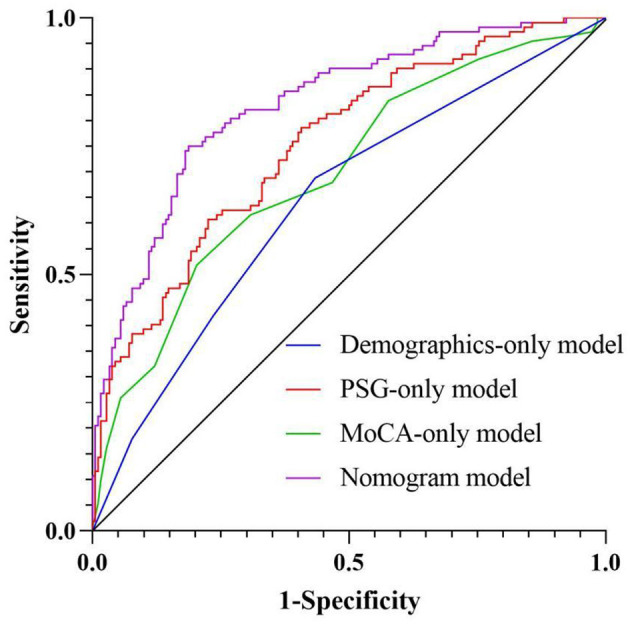
ROC curve analysis of related factors.

We have conducted systematic comparisons between single-dimension models and our full multidimensional nomogram. The Delong test results demonstrate statistically superior performance of our integrated model (AUC = 0.832) vs. all single-dimension approaches (AUC range = 0.642–0.754; all *P* < 0.001), with particular improvements in specificity (81.3 vs. 56.6–79.7%) and sensitivity (75.0 vs. 51.8–68.8%). These findings, now detailed in a new results subsection and Supplementary Table 1, robustly validate our multidimensional approach's clinical advantage in olfactory dysfunction prediction for OSAHS patients (Figure 6 and Table 5).

**Table 5:** The results of the Delong.

**Variable**	**AUC**	**95% CI**	***P*-value**	**Sensitivity**	**Specificity**	**Delong *Z***	***P*-value**
Demographics-only model	0.642	0.576–0.707	< 0.001	68.8	56.6	5.751	<0.001
PSG-only model	0.754	0.697–0.810	<0.001	60.7	77.5	3.622	<0.001
MoCA-only model	0.698	0.636–0.760	<0.001	51.8	79.7	4.527	<0.001
Nomogram model	0.832	0.784–0.880	<0.001	75.0	81.3		

The results of the Delong test show that the predictive value of the Nomogram model is statistically significantly different from that of the other three models.

**Figure 7:**
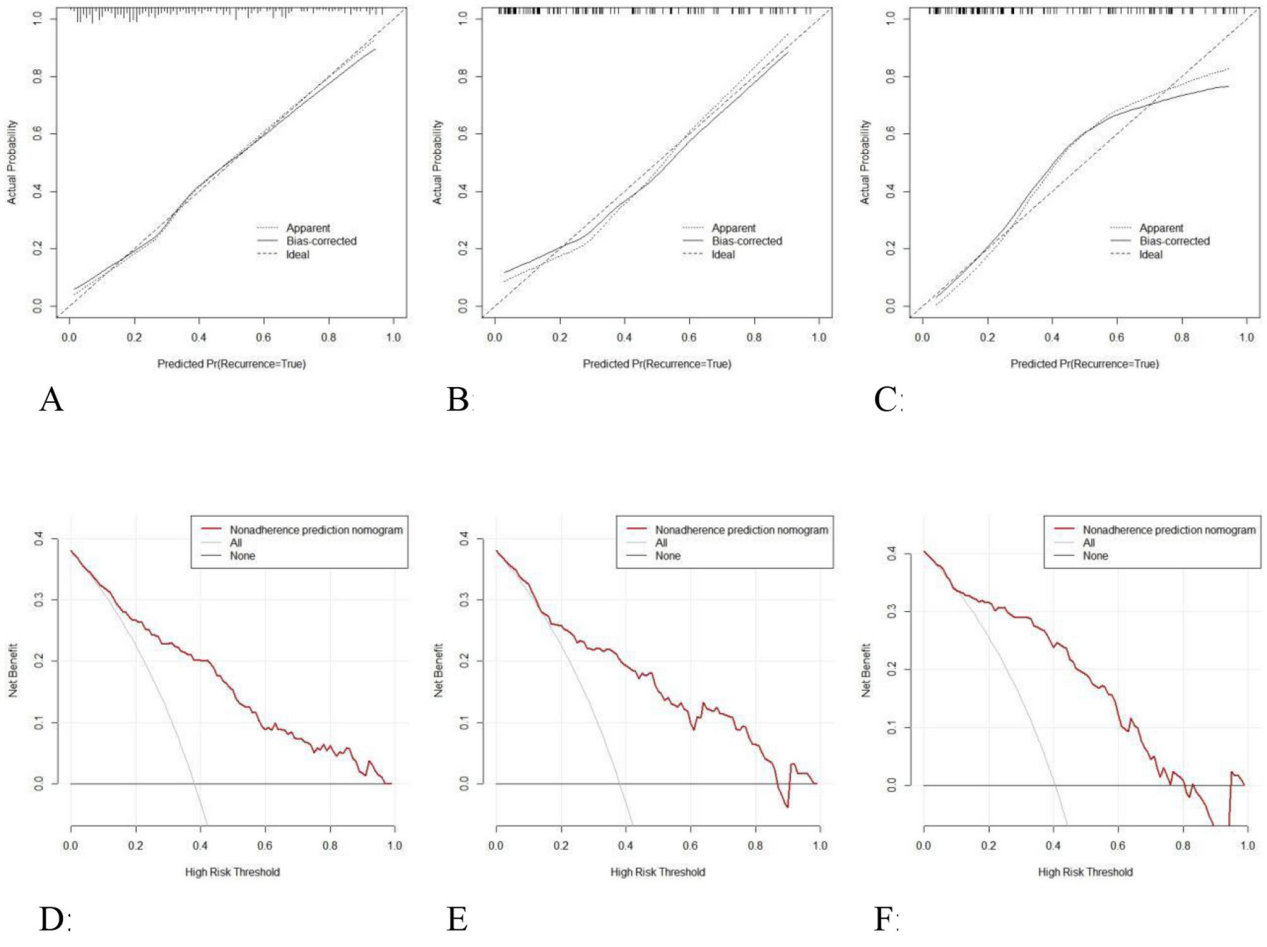
Calibration curves of the model in the training set **(A)**, internal validation set **(B)**, and external validation set **(C)**; Decision curve analysis of the model in the training set **(D)**, internal validation set **(E)**, and external validation set **(F)**.

Calibration curve analysis demonstrates that the model's predicted probability closely aligns with the observed probability (Figures 7A–C). The calibration curve for the training set has a slope of 0.89 (Hosmer–Lemeshow test: χ^2^ = 7.24, *P* = 0.41). The prediction error in the internal validation set following Bootstrap correction amounted to 0.023 (95% CI: 0.015–0.035), and the calibration intercept for the external validation set amounted to −0.018 (suggesting a slight underestimation risk, but the 95% CI included 0), verifying the cross-center applicability of the model.

DCA was employed to assess the clinical utility of the diagnostic graph model through net benefit analysis (Figures 7D–F). The results demonstrate that across a threshold probability range of 6%−97%, the model's net benefit exceeds that of the “full intervention” and “full no intervention” strategies, with the maximum net benefit value reaching 0.38 (equivalent to 38 unnecessary interventions per 100 patients). The net benefit curve remained consistently above the reference line across a threshold range of 13%−85% in the internal validation set, with the clinical application range encompassing moderate to high-risk populations. The net benefit remained stable across a threshold range of 10%−76% in the external validation set, the net benefit was stable, with an average net benefit rate of 29.7%, demonstrating the model's decision-making utility across diverse clinical settings. The model demonstrates particular clinical utility within the 24.2%−78.8% risk threshold range, which corresponds to established intervention points for olfactory rehabilitation in OSAHS patients. Clinicians may consider initiating basic olfactory monitoring at 60% risk and intensive interventions above 60% risk probability.

## Discussion

Based on multicenter retrospective data, this study constructed a nomogram prediction model for olfactory dysfunction in OSAHS patients, incorporating predictive factors including demographic characteristics (male gender, age), polysomnographic parameters (AHI, N3, REM, TS90%), and cognitive function (MoCA). With 546 OSAHS patients enrolled (420 in the training/internal validation sets and 126 in the external validation set), our study represents one of the largest investigations of olfactory dysfunction in OSAHS to date. This sample size substantially exceeds those of previous studies in this field, including Günbey et al. (11) (*n* = 87), Karakurt et al. (3) (*n* = 68), and Liu et al. (9) (*n* = 102), providing greater statistical power for our analyses. Our multicenter approach incorporating both internal and external validation sets from geographically distinct medical centers represents a key strength. This design enhances the generalizability of our findings beyond single-center studies and reduces the risk of region-specific biases. The consistent model performance across validation sets supports the robustness of our nomogram (4, 8). Additional strengths include: (1) comprehensive assessment incorporating PSG parameters, cognitive function, and demographic factors; (2) standardized olfactory evaluation using Sniffin' Sticks tests administered by certified technicians; (3) rigorous statistical methods including LASSO regression with cross-validation; and (4) clinical utility assessment through decision curve analysis. The model providing a visual tool for early clinical identification of high-risk populations.

Numerous previous studies in the general population have demonstrated that women exhibit superior olfactory sensitivity compared to men (21). Our study shares the common focus on predicting olfactory dysfunction in OSAHS patients with Dong et al.'s (8) important work, our research makes several novel contributions. First, we incorporated a more comprehensive panel of PSG parameters including N3 and REM% sleep stages, which were not considered in Dong et al.'s model but demonstrate significant predictive value in our analysis (OR = 0.843 and 0.925, respectively, *P* < 0.01). Second, our multicenter design with rigorous internal and external validation (total *n* = 546 across two medical centers) provides stronger evidence for clinical generalizability compared to their single-center study (*n* = 402). Third, we developed a practical nomogram tool that uniquely integrates cognitive assessment (MoCA scores) with polysomnographic parameters, directly addressing the implementation gap noted in their conclusions. Finally, our LASSO-based variable selection approach offers distinct methodological advantages in handling the inherent multicollinearity among sleep parameters compared to their traditional logistic regression method, as evidenced by our model's robust performance across validation sets (AUC 0.832–0.852). These advancements collectively provide clinicians with a more comprehensive and reliable tool for early identification of high-risk OSAHS patients. Research indicates that female infants demonstrate greater interest in olfactory stimuli from birth. Women's enhanced olfactory capability may be attributed to early endocrine-related influences on olfactory processing regions in the human brain, combined with hormonal mechanisms affecting olfactory perception in adulthood (22). Additional studies suggest that threshold sensitivity to certain odors may fluctuate with menstrual cycles and be influenced by variations in female hormones (23). However, Kern's study of individuals aged 62–90 years found no gender differences in olfactory ability (24). This may reflect age-related deterioration of olfactory function in both genders, leading to diminished differences. As our study did not stratify OSAHS patients by age and gender subgroups, we cannot determine whether gender differences in olfactory dysfunction persist among elderly OSAHS patients. Future research should conduct gender- and age-stratified analyses to further characterize olfactory dysfunction patterns.

Dong et al. (8) identified age as an independent risk factor for olfactory dysfunction in OSAHS patients. Our results show significantly increased risk of olfactory dysfunction in OSAHS patients aged ≥60 years, consistent with previous findings. Animal studies demonstrate significantly increased expression of apoptotic genes in the olfactory system of aged rats, promoting olfactory receptor neuron death (25). Previous research confirms that the prevalence of olfactory dysfunction increases progressively with age (26). Age-related olfactory impairment may result from reduced numbers of olfactory receptor neurons and olfactory bulb fibers, cortical changes, alterations in cribriform plate porosity, or changes in mucus composition (27). Hypoxia in OSAHS patients may accelerate aging of the olfactory system. Declining cerebral blood flow autoregulation in elderly patients, combined with intermittent hypoxia from sleep-disordered breathing, may precipitate premature olfactory dysfunction. While continuous positive airway pressure therapy has been shown to improve olfactory dysfunction in OSAHS patients (28), whether this improvement varies by age remains unknown. Future studies should compare therapeutic outcomes between OSAHS patients with olfactory dysfunction below and above 60 years to determine potential age-related differences in treatment response.

Our study identified AHI, N3, REM, and TS90% as independent risk factors for olfactory dysfunction. Existing research has established that AHI, being the primary diagnostic metric for OSAHS, shows significant correlation with olfactory impairment (9). Günbey et al. (11) demonstrated that patients with severe OSAHS (AHI ≥ 30 events/h) exhibited lower olfactory discrimination scores compared to mild cases. Our findings reveal a dose-dependent relationship between respiratory event frequency and olfactory dysfunction, with each 1-unit AHI increase elevating the risk by 11.3% (OR = 1.113). Hence, a dose-effect relationship is observed between respiratory events frequency and olfactory function impairment. Karakurt et al. demonstrated that TS90% showed a negative correlation with the olfactory threshold using PSG parameters. In this study, the risk association was quantified with an odds ratio of 1.306. In this study, the risk association was quantified with an odds ratio of 1.306, and a 1% increase in chronic hypoxia load was associated with a 30.6% higher risk of olfactory disorders (3). Patients with OSAHS are prone to sleep fragmentation and daytime sleepiness resulting from nocturnal hypoxia. Regarding sleep structure parameters, Karakurt et al. demonstrated a significant positive correlation between REM sleep proportion and olfactory scores, while the impact of N3 stage sleep remained underexplored (3). This study demonstrates that a 1% increase in deep sleep is associated with a 15.7% reduction in risk, whereas a 1% increase in REM sleep is associated with a 7% reduction in risk. This finding aligns with findings from animal studies demonstrating that NREM slow-wave sleep facilitates olfactory bulb nerve regeneration, while REM sleep enhances odor memory consolidation through distinct molecular mechanisms (25). Physiological changes occurring during sleep are classified into distinct sleep stages based on electroencephalogram (EEG) frequency patterns. Sleep stages are classified into NREM and REM stages, with the NREM stage further subdivided into N1, N2, and N3 stages based on distinct physiological characteristics. Following the N3 phase of NREM, the sleep cycle transitions rapidly into the REM stage. The pulsed secretion of growth hormone during the N3 stage facilitates the repair of olfactory sheath cells, whereas cholinergic activation in the REM stage sustains the synaptic plasticity in the olfactory cortex (27).

The study findings demonstrate that the MoCA score is an independent predictor of olfactory disorders. Previous studies have demonstrated the existence of a comorbidity between cognitive decline and olfactory disorders in patients with OSAHS, although the underlying mechanisms remain poorly understood (29). Günbey et al. (11) demonstrated that attention deficit and impaired executive function in patients with OSAHS may contribute to reduced odor discrimination scores by impairing the prefrontal cortex's capacity to integrate olfactory information. The MoCA score, as a multi-dimensional tool for assessing cognitive function (encompassing subdomains such as memory, executive function, and attention), is significantly associated with a lower risk of olfactory disorders, suggesting that microstructural damage to the prefrontal cortex and hippocampal structures attributable to long-term intermittent hypoxemia and disrupted sleep architecture in patients with OSAHS may be the key pathological basis (30). Animal experiments have shown that chronic intermittent hypoxia induces neuronal apoptosis in the hippocampal and downregulates olfactory marker protein expression in the olfactory bulb, whereas oxidative stress in the prefrontal cortex may impair odor memory encoding through suppression of synaptic plasticity (7). In addition, growth hormone deficiency attributable to disrupted sleep architecture (such as decreased N3 sleep) may further impair the regeneration of olfactory sheath cells, contributing to a self-perpetuating cycle of “hypoxia–cognitive decline–olfactory disorder.” The nomogram of this study shows that for patients with a MoCA score of ≤ 24, even if the AHI is < 30 times/h, the probability of olfactory disorder still reaches 65%. Thus, cognitive assessment can serve as a valuable complement to conventional PSG parameters.

While our nomogram demonstrates strong predictive performance, its implementation in resource-limited settings warrants careful consideration due to its inclusion of PSG parameters (particularly N3 and REM% sleep stages) that require overnight monitoring. For clinical settings where polysomnography is unavailable, we propose several practical adaptation strategies: (1) the demographic (age, gender) and cognitive (MoCA score) components can serve as an initial screening tool, maintaining reasonable discrimination; (2) this two-parameter approach could identify high-risk patients warranting referral for comprehensive evaluation; (3) portable sleep monitoring devices may provide surrogate measures of sleep architecture; (4) clinical prediction rules combining anthropometric measures (neck circumference, BMI) with symptom scores could approximate PSG parameters.

In conclusion, the nomogram model developed in this study can estimate the risk probability of olfactory disorders among patients with OSAHS. Based on the nomogram-derived risk scores, we established a three-tier risk stratification system with corresponding clinical management protocols: (1) low-risk patients (score ≤ 100, olfactory dysfunction probability < 30%) should receive annual olfactory screening, standard CPAP therapy, and basic nutritional counseling; (2) intermediate-risk patients (score 100–150, probability 30%−60%) require biannual olfactory monitoring, optimized CPAP therapy, cognitive assessment every 6 months, and consideration of olfactory training; (3) high-risk patients (score > 150, probability > 60%) need quarterly olfactory assessments, strict CPAP compliance monitoring, olfactory rehabilitation therapy, and neurological consultation. This graduated approach ensures appropriate clinical interventions tailored to each patient's risk level while optimizing resource allocation. For example, a 62-year-old male patient exhibited an AHI of 45 events/h, N3 sleep percentage 4%, REM sleep percentage of 7%, TS90 of 8%, and a MoCA score of 22 The patient's nomogram-derived score was 185 points, calculated as follows: male (30 points)+ age ≥60 years old (25 points)+ AHI = 45 (40 points) + N3% = 4 (20 points) + REM% = 7 (15 points) + TS90% = 8 (25 points) + MoCA = 22 (30 points). The corresponding probability axis shows that the risk of olfactory disorder is 78%. It is recommended to prioritize continuous positive airway pressure ventilation treatment and to monitor olfactory function. Therefore, this study combines PSG parameters with cognitive assessment to develop a prediction model, overcoming the limitations of unidimensional analysis. The LASSO algorithm is employed to address multicollinearity and enhance the robustness of the model. The external validation set demonstrates cross-center generalizability.

It should be acknowledged that there are several limitations: (1) the retrospective design cannot determine causal relationships. We did not assess potential mechanistic biomarkers (such as inflammatory markers); conductive and sensory olfactory dysfunction were not distinguished. Although our sample size was larger than previous studies, it was only from two centers in Sichuan Province, which may limit its generalizability to other populations. Future prospective multicenter studies incorporating biomarker assessment will help address these limitations. Future research should include nasal resistance measurement and imaging techniques to distinguish between conductive and sensory components, which may further improve predictive accuracy. (2) Future studies should explore predictive models for specific age groups, especially for the pediatric population, as their unique pathophysiological mechanisms may require different predictive methods. Developing such models requires age-appropriate olfactory testing protocols, adjusted interpretation of polysomnography parameters based on age, and validation in dedicated pediatric cohorts. (3) Future studies can conduct large-scale sample research to further explore the interaction of comorbidities. (4) Although our nomogram provides valuable baseline risk stratification, the cross-sectional nature of this study cannot assess the olfactory recovery trajectory after continuous positive airway pressure (CPAP) treatment. Future studies should establish dedicated longitudinal cohorts and conduct continuous olfactory assessments to comprehensively capture treatment responses. (5) Although our exclusion criteria aimed to minimize confounding factors from other causes of olfactory dysfunction, future studies with larger sample sizes can consider competing risk analysis to further improve the specificity of predicting olfactory impairment related to obstructive sleep apnea-hypopnea syndrome (OSAHS). Although this study demonstrated clinical utility, a formal cost-benefit analysis should be conducted in implementation studies, including the incremental cost per quality-adjusted life year (QALY) gained from early olfactory intervention, long-term healthcare savings achieved through prevention of complications, and comparison of continuous olfactory testing strategies. (6) Although the total score of the Montreal Cognitive Assessment (MoCA) can effectively reflect the overall cognitive impairment caused by olfactory dysfunction related to obstructive sleep apnea-hypopnea syndrome (OSAHS), our subsequent related research conducted detailed analyses of the relationship between specific cognitive domains and olfaction through extended neuropsychological tests. (7) While LASSO effectively handles multicollinearity, the high VIF values for certain sleep parameters suggest these measures capture overlapping physiological information. Future studies might consider dimension reduction techniques prior to modeling. (8) As a cross-sectional study, our findings can only demonstrate statistical associations between variables and the outcome. The temporal sequence between exposure and outcome cannot be established, and thus causal inferences should be avoided. Future longitudinal studies are needed to verify these observed relationships.

## Conclusion

The study has successfully developed a prediction model incorporating gender, age, AHI, N3, REM, TS90% and MoCA scores that demonstrates excellent capability in identifying high-risk populations for OSAHS-associated olfactory dysfunction. The model exhibits strong discriminative power and calibration accuracy, and offering clinicians a reliable, non-invasive assessment tool to facilitate early intervention strategies.

## Data Availability

The original contributions presented in the study are included in the article/Supplementary material, further inquiries can be directed to the corresponding author.
