# Supplementary material for: Nomogram for predicting early olfactory dysfunction in obstructive sleep apnea-hypopnea syndrome: a multicenter-based study
Source: Front Neurol. 2025 Sep 17;16:1635012. doi: 10.3389/fneur.2025.1635012 (PMC12485232; doi:10.3389/fneur.2025.1635012)
Supplement: Supplementary file 1 [file Data_Sheet_1.docx]

Table S1 Comprehensive VIF analysis

| \| Variable \| \| --- \| | VIF | 项目 | VIF |
| --- | --- | --- | --- | --- |
| Gender | 1.083 | SE | 10.116 |
| Age | 1.095 | TS90 | 4.081 |
| Education years | 1.062 | LSaO2 | 1.081 |
| BMI | 4.05 | WASO | 13.068 |
| Smoking | 1.038 | N1 | 5.089 |
| Alcohol | 1.054 | N2 | 1.078 |
| Hypertension | 3.082 | N3 | 6.09 |
| Diabetes | 1.052 | REM | 7.086 |
| Hyperlipidemia | 2.085 | ESS | 21.089 |
| Family OSAHS | 1.081 | ISI | 1.075 |
| AHI | 1.104 | MoCA | 1.082 |
| TST | 6.066 |  |  |
